# Study of an Online Monitoring Adaptive System for an Injection Molding Process Based on a Nozzle Pressure Curve

**DOI:** 10.3390/polym13040555

**Published:** 2021-02-13

**Authors:** Jia-Chen Fan-Jiang, Chi-Wei Su, Guan-Yan Liou, Sheng-Jye Hwang, Huei-Huang Lee, Hsin-Shu Peng, Hsiao-Yeh Chu

**Affiliations:** 1 Department of Mechanical Engineering, National Cheng Kung University, Tainan 700, Taiwan; adam01172073@gmail.com (J.-C.F.-J.); hyde00155@gmail.com (C.-W.S.); n16094182@gs.ncku.edu.tw (G.-Y.L.); 2 Department of Engineering Science, National Cheng Kung University, Tainan 700, Taiwan; hhlee@mail.ncku.edu.tw; 3 Department of Mechanical and Computer-Aided Engineering, Feng Chia University, Taichung 400, Taiwan; hspeng@fcu.edu.tw; 4 Department of Mechanical Engineering, Kun Shan University, Tainan 700, Taiwan; hsiaoyeh@mail.ksu.edu.tw

**Keywords:** injection molding process, pressure curve, peak pressure, viscosity index, *V*/*P* switchover point, adaptively adjustment system

## Abstract

Injection molding is a popular process for the mass production of polymer products, but due to the characteristics of the injection process, there are many factors that will affect the product quality during the long fabrication processes. In this study, an adaptive adjustment system was developed by C++ programming to adjust the *V*/*P* switchover point and injection speed during the injection molding process in order to minimize the variation of the product weight. Based on a series of preliminary experiments, it was found that the viscosity index and peak pressure had a strong correlation with the weight of the injection-molded parts. Therefore, the viscosity index and peak pressure are used to guide the adjustment in the presented control system, and only one nozzle pressure sensor is used in the system. The results of the preliminary experiments indicate that the reduction of the packing time and setting enough clamping force can decrease the variation of the injected weight without turning on the adaptive control system; meanwhile, the master pressure curve obtained from the preliminary experiment was used as the control target of the system. With this system, the variation of the product weight and coefficient of variation (*C_V_*) of the product weight can be decreased to 0.21 and 0.05%, respectively.

## 1. Introduction

**Figure 1:**
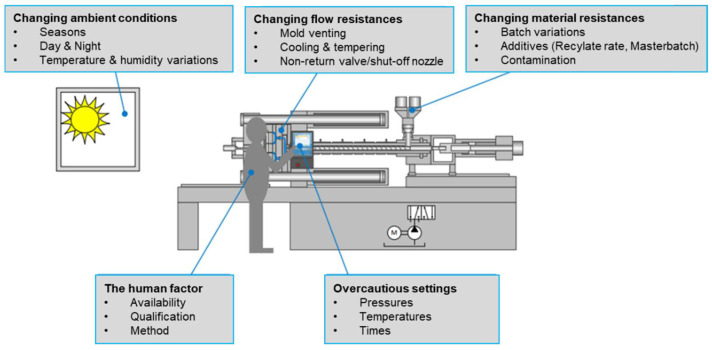
Factors affecting the product weight in the injection molding process [1].

Malinowski [1] mentioned that, during processing, decreasing uncertainty related to product quality was a very important issue for injection molding processing, with environmental factors, plastic flow resistance, material changes, and human factors being considered to be causes of such an uncertainty (shown in Figure 1).

The injection molding process can be divided into seven stages: plasticization, clamping, injection, packing, cooling, demolding and ejecting. Injection and packing are the stages that have been suggested to have the greatest effects on product quality. Kazmer et al. [2] mentioned that changes in the screw position and injection pressure were conditions that could decrease product quality uncertainty. Excluding the switchover position, injection speed is also an important processing parameter for injection molding machines. In the case of polymer materials, the *P*–*V*–*T* (Pressure-Specific Volume–Temperature) relationship is very important because there is a strong relationship between pressure, volume, and temperature, and the specific volume affects the product weight significantly. Using the relationship between pressure and temperature to maintain a specific volume can lead to a constant product weight.

Michaeli and Schreiber [3] used feedback cavity pressure data and the *P*–*V*–*T* relationship of a polymer to control the product quality cycle by cycle. There have been many methods to control injection molding machines; for instance, Chen et al. [4] and Chen et al. [5] have found that the clamping force has a strong relationship with the product weight, then use the clamping as a criterion to adjust the switchover position; additionally, Huang [6] also use the cavity pressure to adjust a better switchover position, and they use the grey prediction to predict the cavity pressure; Chen and Turng [7] use the mold separation and part weight as a criterion for adjusting the parameter.

For a thin plastic product, the injection speed plays a big part, and it can influence the product quality a lot. Pandelidis et al. [8] and Yang et al. [9] have mentioned that they tuned the injection speed by controlling the servo valve, before maintaining the thin plastic product’s quality. Tracking the master curve is a very important method for injection molding; Agrawal et al. [10] built a master curve that was based on the cavity pressure curve in order to maintain the injection speed; then, Zhang et al. [11] used the prediction model of the warpage to control the injection parameter, furthermore maintaining the warpage of the product; following this, Dubay [12] also stabilized the product quality by setting an ideal quality curve as the control benchmark of the predictive control model. 

In this study, the pressure curve is an important basis for judging the product weight. Schiffers [13] used the viscosity index (*VI*) to describe the pressure curve and used it as an indicator of product quality as well as to determine the best switchover position.

Chen et al. [14] developed an adaptive system and used a nozzle pressure sensor to measure and monitor polymer pressure. Nozzle pressure data was also used to calculate the viscosity index. In the experiment, Chen found that the viscosity index had a significant relationship with the product weight, where this relationship could be controlled by the switchover position.

Tsai [15] developed an adaptive system that was dependent on a neural network prediction system and used the injection speed, switchover position, and cavity temperature through the neural network system to predict the pressure curve characteristics. With this system, the variations of the product weight were decreased to 0.14%, and it was proven that the injection speed is an important parameter for the injection process.

In this study, the viscosity index and peak pressure are used to guide the adjustment in the presented control system, and only one nozzle pressure sensor is used in the system. The sensor was placed on the side of the nozzle. The adaptive adjustment control system stabilized the pressure curve and, in turn, stabilized the product weight.

## 2. Methods and Experiment

### 2.1. Injection Molding Process

**Figure 2:**
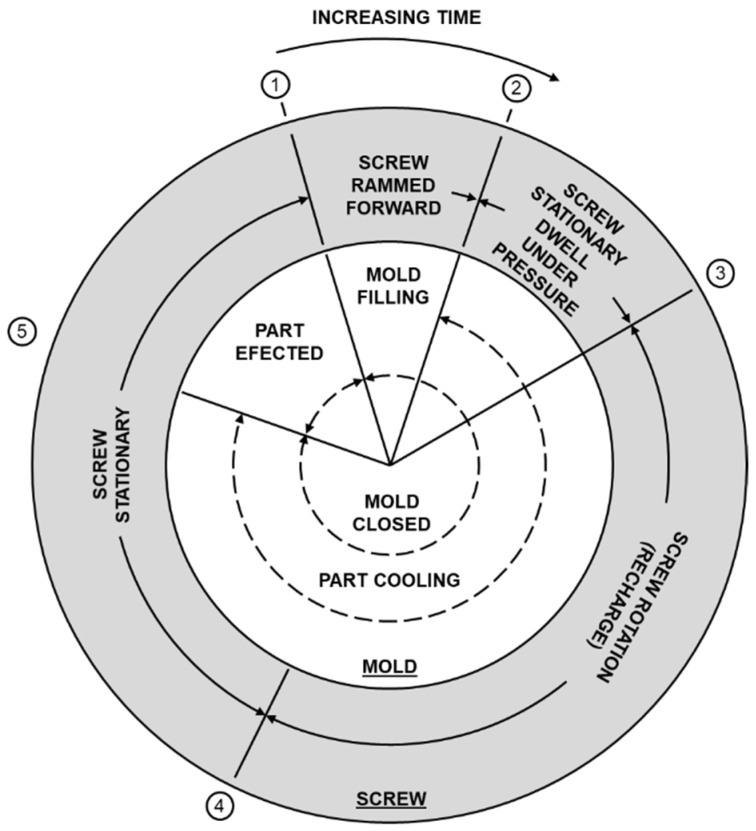
Injection molding process [16].

The process cycle is divided into the following stages, shown in Figure 2:

- 1–2: After closing the mold, the screw moves forward to allow the plastic to fill the cavity.
- 2–3: The screw remains stationary under pressure, and the cooling function begins.
- 3–4: The screw moves back to the initial position to allow the material to fill for the next cycle.
- 4–5: The screw remains in a stationary position to wait for the next mold and stops cooling.
- 5–1: After the mold opens, the product is ejected.

### 2.2. P–V–T Relationship

**Figure 3:**
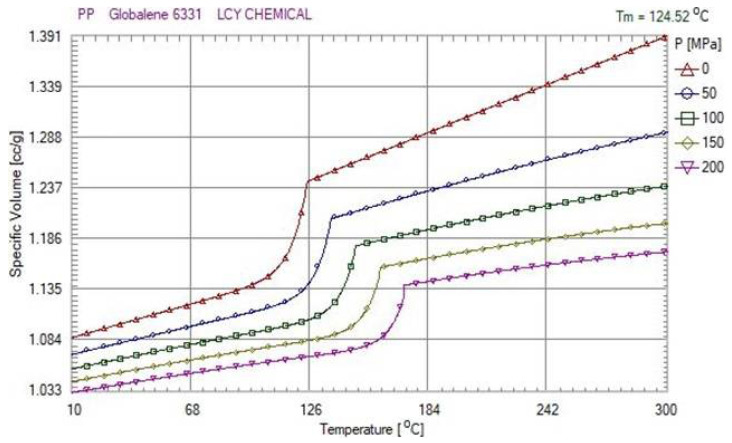
*P*–*V*–*T* (Pressure-Specific Volume-Temperature) Relationship of Polypropylene (PP).

In this study, polypropylene was used as the experimental material. The *P*–*V*–*T* (Pressure-Specific Volume–Temperature) properties of polymers are important for both engineering and polymer physics. At a constant pressure, the specific volume is directly proportional to the melt temperature. Figure 3 presents the *P*–*V*–*T* relationship of polypropylene. This is why determining how to effectively control the injection speed and switchover position in order to stabilize the pressure curve is a very important issue for the injection molding process.

### 2.3. Peak Pressure and Viscosity Index

During the injection process, even when the material is the same, the product quality will be affected by environmental factors and batch factors. Huang [17] used a pressure sensor to capture pressure data and used the feedback data to calculate the peak pressure, viscosity index, energy index, and pressure gradient. Based on the experimental results, it was found that with increases in the injection speed, these four parameters will exhibit the same trend as that of the product weight, but only the peak pressure, viscosity index, and energy index have a strong relationship with the product weight. Even though the energy index has a strong relationship with the product weight, the energy index capture and calculation process are more complex than the viscosity index, making it necessary to describe the pressure curve by obtaining the screw position, whereas only a pressure sensor is required in order to use the viscosity index to describe the pressure curve. 

**Figure 4:**
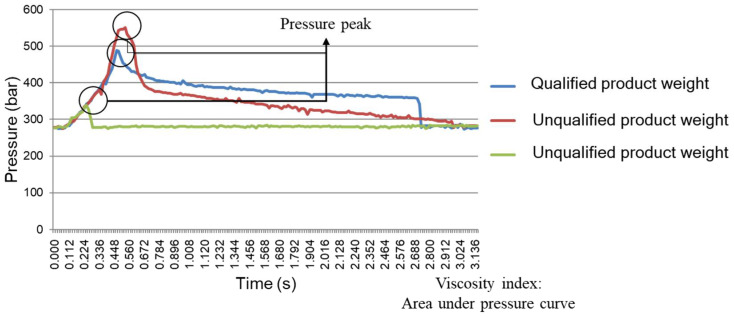
Product quality with different pressure curves.

In this study, the viscosity index and peak pressure comprised the adjust criteria for the system. Figure 4 shows that a pressure curve with a higher peak pressure and larger area will indicate a greater product weight; otherwise, the product weight will be lighter. The peak on the pressure curve is the peak pressure *P_peak_*.

To describe the different properties of polymers during the injection molding process, the viscosity index is used in this study, which is an integral of a pressure curve. Both the viscosity index and peak pressure can be used to describe the pressure curve.

(1) $$VI=\int_{t_{injection_{start}}}^{t_{packing_{end}}}P_{Melt}\left(t\right)dt$$

- Where *VI* is a viscosity index, *t* is time, and *P_Melt_* is the melt pressure, and where the injection start is a start signal, and the packing end is the packing end signal.
- Both the viscosity index and peak pressure can be indicators of the product weight, and these two parameters can be used to determine better injection speeds and switchover positions.
- Through the preliminary experiment, it was clearly observed that the peak pressure and viscosity index were directly proportional to the product weight. The results of the preliminary experiment were used to obtain the master curve in order to implement it into the adaptive adjustment system.

### 2.4. Adaptive Process Control

Adaptive control is the control method that must adapt to a controlled system with parameters that vary or are initially uncertain. The basic concept of adaptive control is that the controller uses the measured input signal to regulate the next output, and the regulated output changes the next input. Thus, adaptive control can also be regarded as the control system of instantaneous parameter regulation.

## 3. Experimental Section

### 3.1. Material

The study chose polypropylene (PP) for the experiment because it is a common material that is used in injection molded parts. The molding material used for the experiment was polypropylene Globalene 6331, which was fabricated using an injection molding process by using LCY GROUP (Li Changrong Chemical, Co. Ltd.; Taipei City, Taiwan). The material properties are shown in Table 1.

### 3.2. Equipments

**Figure 5:**
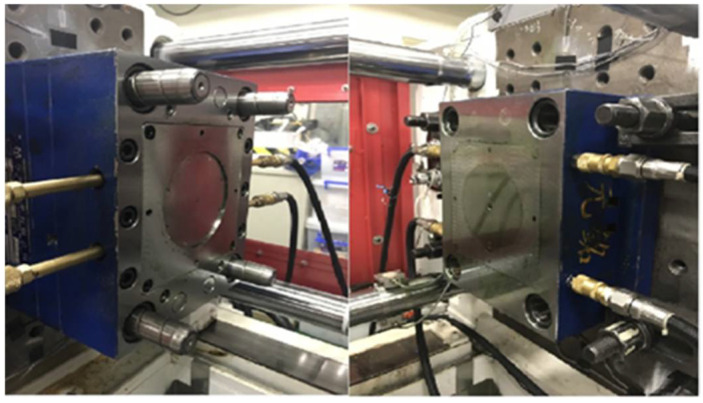
The mold of the thin disk sample.

General purpose polypropylene was used for the injection molding of a thin plastic disk (see Figure 5). A pressure sensor with a sensitivity of 3.3 mV/bar (Dynisco, PT4655XL, Dynisco, MA, USA) was mounted on the nozzle to measure the melt pressure. The pressure sensor specifications are shown in Table 2. A data acquisition module (USB-4716, Advantech Co., Ltd., Taipei, Taiwan) with a high sampling rate (20 kHz) was used to obtain the nozzle pressure data. The data acquisition module specifications are shown in Table 3. A 60-ton injection molding machine (CLF-60TX, Chuan Lih Fa Co., Ltd., Tainan, Taiwan) with a machine controller (MIRLE automation corporation) was used to fabricate samples under a variety of process parameters. The maximum injection rate was 115 (cm^3^/s), the maximum injection pressure was 2951 (kg/cm^2^), and the screw diameter was 30 mm. The injection molding machine specifications are shown in Table 4.

## 4. Preliminary Experiments, Master Curve, and Adaptive Adjustment Experiments

### 4.1. The Experiments with Eight Seconds of Packing Time

The experiment parameters in Table 5 were used to verify the relationship between the injection speed, switchover position, viscosity index, peak pressure, and product weight. At the same time, we observed that if the packing time was too long, it would cause the product weight to become unstable. Therefore, in the next group of experiments, the packing time was set at three seconds. The detailed results are in Section 5.1.

### 4.2. The Experiments for Three Seconds of Packing Time with Different Amounts of Clamping Force

The experiment parameters for three seconds of packing time with different amounts of clamping force are shown in Table 6. In the experiments, it could be observed that the peak pressure and the viscosity index followed the same trend as the product weight. At the same time, with the parameter of three seconds of packing time and 40 tons of clamping force, the variations of the product weight were more stable than with eight seconds of packing time. The detailed results are in Section 5.2.

### 4.3. The Full-Factorial Experiments for Three Seconds of Packing Time

The experiments combined the results of Table 5 and Table 6. The experiments were set at three seconds of packing time, 40 tons of clamping force, and designed a full-factorial experiment in Table 7. The results showed that the peak pressure, viscosity index, and product weight had the same trend and that the variations of the product weight clearly decreased. Therefore, we used the experimental results as the judgment criterion for the adaptive control system. The detailed results are in Section 5.3.

### 4.4. Master Curve

**Figure 6:**
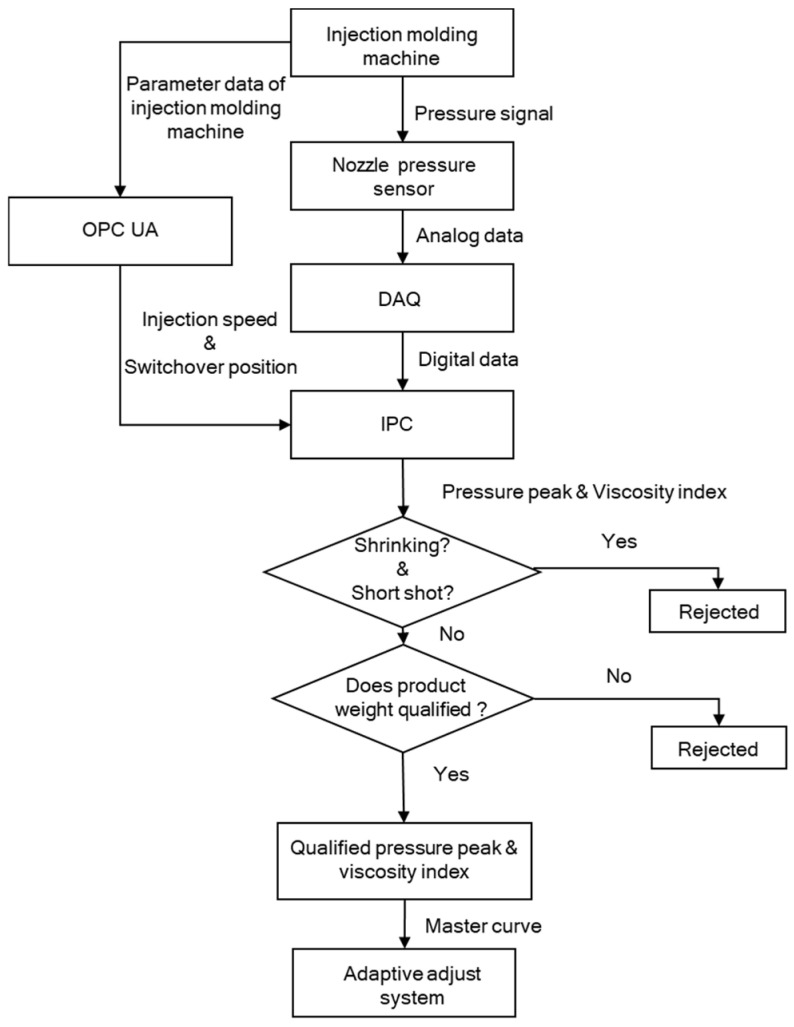
Decision flow chart of the master curve.

The flow chart shown in Figure 6 provides the criteria for judging whether the pressure peaks and viscosity indexes can be put into the adaptive control system, where the verified pressure peaks and viscosity indexes will be put into the master curve, and the master curve will be implemented in the adaptive adjust system as a control algorithm.

### 4.5. The Adaptive Control System Experiments

**Figure 7:**
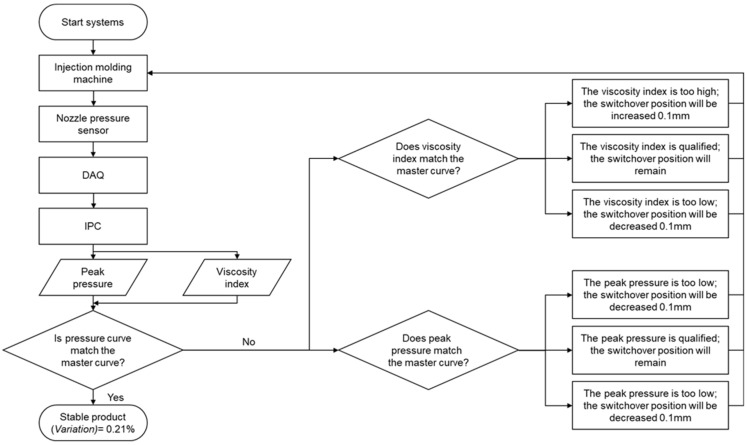
Flow chart of the control system.

An adaptive adjustment system was developed in this study. The flow chart of the system is presented in Figure 7. The system uses the feedback pressure data from DAQ, and then uses the pressure data signal from the injection start to the packing end to calculate the viscosity index and peak pressure. The master curves derived from the preliminary experiments were used as the criteria by which to verify the product quality, where if the quality was verified, the process continued, and if the quality was unverified, the adaptive adjustment system stabilized the product quality by adjusting the injection speed and the switchover position before continuing the production process.

Two experiments were conducted in this study: the one conducted with the parameters described in Table 8 was without the adaptive adjustment system, and the other conducted with the parameters shown in Table 9 was with the system, and both performed 100 cycles.

## 5. Results

### 5.1. The Results of The Experiments with Eight Seconds of Packing Time

**Figure 8:**
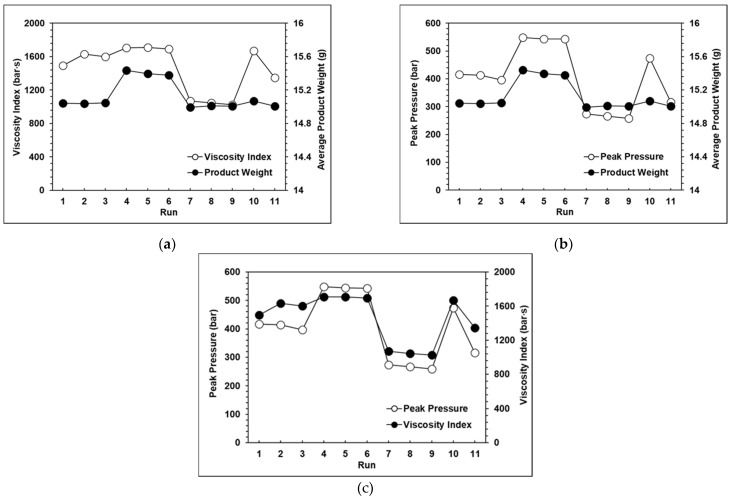
(**a**) Relationship between the product weight and the viscosity index with eight seconds of packing time; (**b**) Relationship between the product weight and peak pressure with eight seconds of packing time; (**c**) Relationship between the viscosity index and peak pressure with eight seconds of packing time.

**Figure 9:**
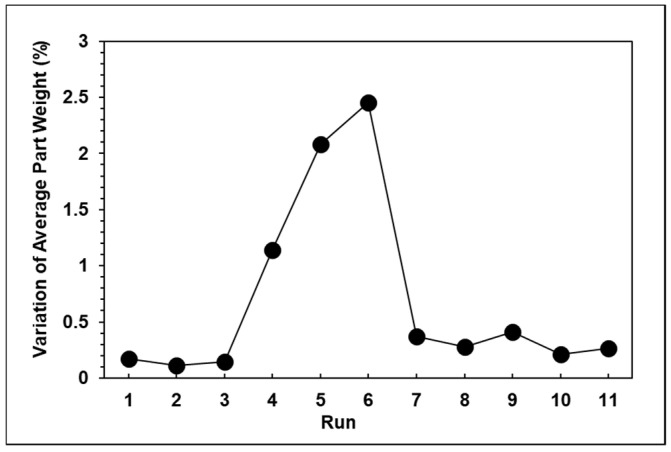
Variations in the average product weight with eight seconds of packing time.

The results of the experiments proved that with a change in the injection speed and switchover position, the peak pressure, viscosity index, and product weight also changed significantly. The relationships among the peak pressure, viscosity index, and product weight can be clearly seen in Figure 8a–c, which verifies that the product weight has a very strong proportional relationship with the viscosity index and peak pressure. However, with the exception of the standard groups 1, 2, and 3, Figure 9 shows that the variations in the average product weight for the other parameters are outside the appropriate range. Using the experimental parameters, it was verified that if the packing time was too long, this would cause the product weight to become unstable, so in the next group of experiments, the packing time was set at three seconds. Here, the variations in product weight were used as an indicator to determine whether the product weight remained stable. The function of the variation in product weight is as follows:

(2) $$Variation=\;\frac{V_{\max}-V_{\min}}{V_{ave}}\times 100\%$$

### 5.2. The Results of the Experiments for Three Seconds of Packing Time with Different Amounts of Clamping Force

**Figure 10:**
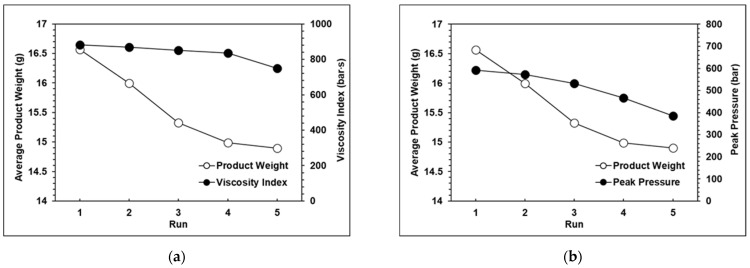
(**a**) Relationship between the product weight and the viscosity index with 20 tons of clamping force; (**b**) Relationship between the product weight and the peak pressure with 20 tons of clamping force.

**Figure 11:**
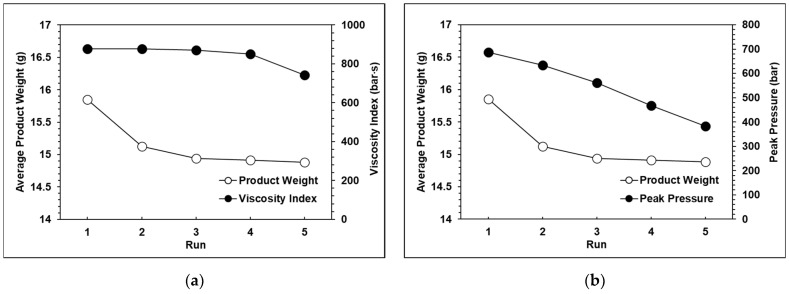
(**a**) Relationship between the product weight and the viscosity index with 30 tons of clamping force; (**b**) RelaTable 30 tons of clamping force.

**Figure 12:**
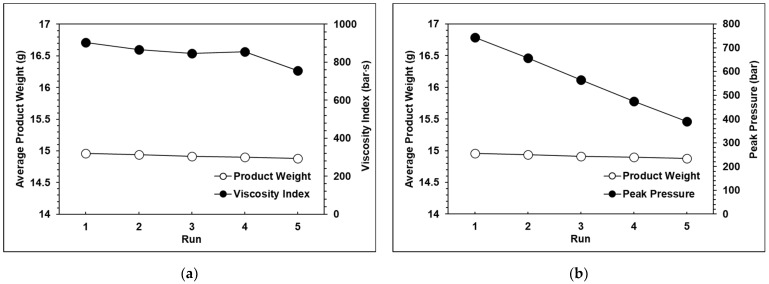
(**a**) Relationship between the product weight and the viscosity index with 40 tons of clamping force; (**b**) Relationship between the product weight and the peak pressure with 40 tons of clamping force.

**Figure 13:**
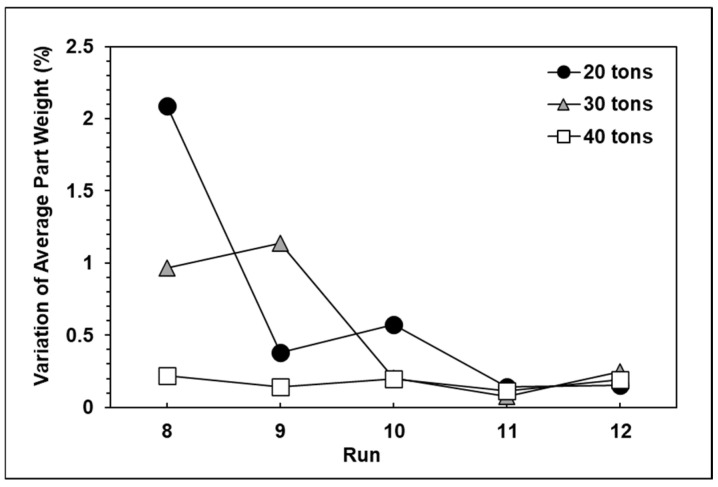
Variations in product weight under different amounts of clamping force.

The experimental results for each group are shown in Figure 10, Figure 11 and Figure 12. It can be observed that the peak pressure and the viscosity index follow the same trend as the product weight. Figure 13 shows that with three seconds of packing and 40 tons of clamping force, the variations of product weight were more stable than with 8 s of packing.

### 5.3. The Results of the Full-Factorial Experiments for Three Seconds of Packing Time

**Figure 14:**
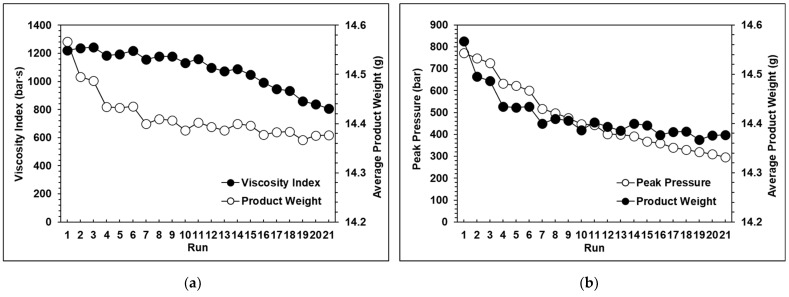

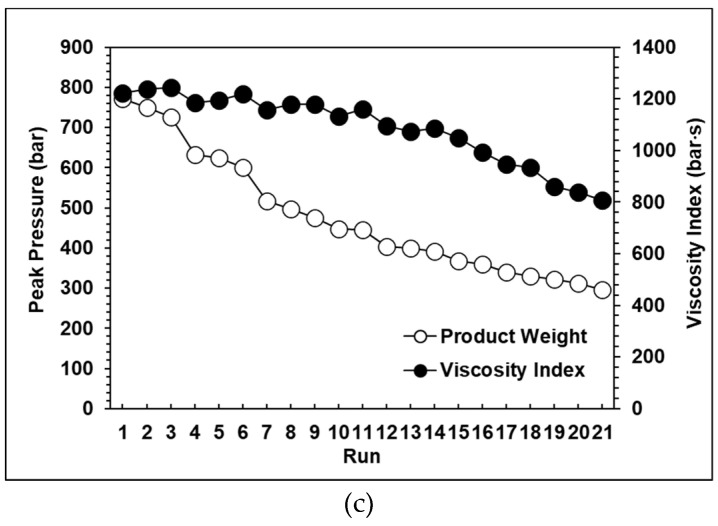
(**a**) Relationship between the product weight and peak pressure after three seconds of packing time; (**b**) Relationship between the product weight and the viscosity index after three seconds of packing time; (**c**) Relationship between the viscosity index and the peak pressure after three seconds of packing time.

**Figure 15:**
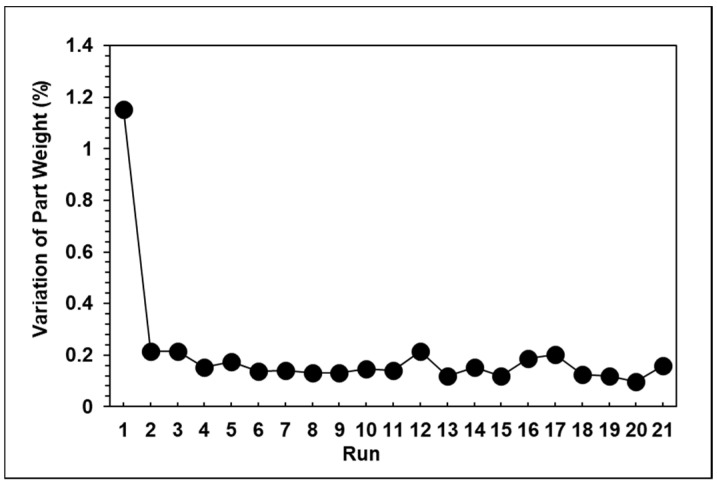
Variations in product weight after three seconds of packing time.

Based on Figure 14, the results show that the peak pressure, viscosity index, and product weight exhibited the same trend. Figure 15 shows that the variations of the product weight clearly decreased when compared to the results after eight seconds of packing.

### 5.4. The Results of the Experiments with the Adaptive Control System

**Figure 16:**
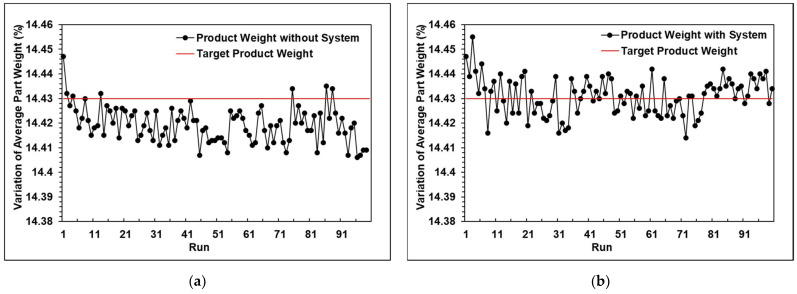
(**a**) Performance without the adaptive adjustment system; (**b**) Performance with the adaptive adjustment system.

**Figure 17:**
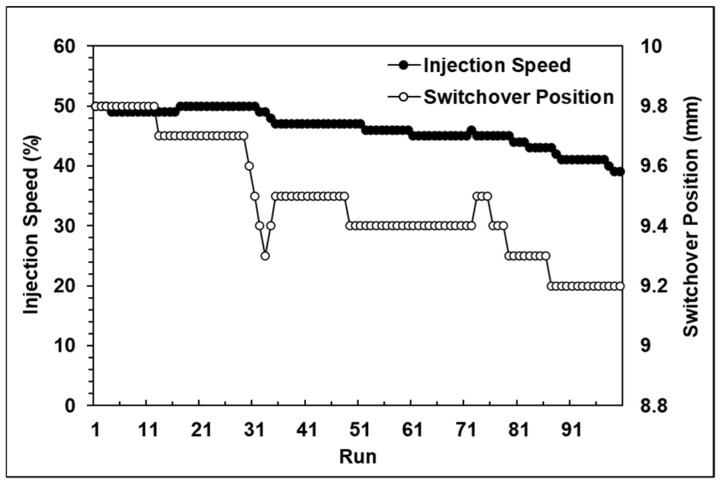
Adjustment process using the adaptive adjustment system.

The experiment without the system was conducted using stable parameters. Figure 16a shows the first 50 cycles without the proposed system, where the product weight was stable. However, after reaching 70 cycles, the product weight trend gradually became lighter, and the weight fluctuations became very intense. The experiment with the adaptive adjustment system was conducted with unstable initial parameters. Figure 16b shows the adjustment process when utilizing the adaptive adjustment system. Figure 17 proves that the system can stabilize the product quality and follow the target value by adjusting the correct parameter.

With the function (2), the variation of the product weight is 0.21%, whereas without the system it was 0.39%. Furthermore, with the functions (3) and (4), the standard deviation and the coefficient of variation can be calculated, where “*σ*” is the standard deviation, “*x*” is the measured weight of each mold product, “*μ*” is the target of the product weight, n is the total amount of mold, and “*C_v_*” is the coefficient of variation. The coefficient of variation of the product weight was 0.05%, whereas without the system it was 0.20%. The results indicate that the process with the system is more stable than the process without the system.

(3) $$\sigma =\sqrt{\frac{\sum_{i=1}^{n}{\left(x_{i}-\mu \right)}^{2}}{n}}$$

(4) $$c_{\upsilon }=\frac{\sigma }{\mu }\times 100\%$$

## 6. Conclusions

In this study, C++ programming language was used to develop an adaptive adjustment system that only used a nozzle pressure sensor to monitor extracted pressure data. According to the *P*–*V*–*T* relationship for polymer characteristics, as soon as the pressure was stabilized during the injection molding process, the weight of the injected parts could also be consistently maintained.

A preliminary experiment was conducted to show the proportional relationships between the peak pressure, viscosity index, and product weight, after which this relationship was used to obtain the master curve that could be implemented in the adaptive adjustment system. Furthermore, the results of the preliminary experiments reveal that more appropriate process parameter settings (i.e., packing time, clamping force) enable the minimization of the variation of injection molded parts before carrying out the adaptive adjustment system.

With this system, the product weight was more stable than for the process without such a system, with the variation of the product weight and the coefficient of variation of the product weight decreasing to 0.21% and 0.05%, respectively. Based on the comparative results, the validity of the proposed system was successfully verified.

## Figures and Tables

**Table 1:** Polypropylene properties [18].

Globalene 6331 Polypropylene Homopolymer			
General Properties	Test Methods	Unit	Values
Melt flow rate (230 °C)	ASTM D1238	g/10 min	14.5
Density	ASTM D792	g/cm^3^	0.904
Elongation at yield	ASTM D638	%	9
Elongation at break	ASTM D638	%	83
Tensile strength at yield	ASTM D638	kg/cm^2^	370
Flexural modulus	ASTM D790	kg/cm^2^	17,500
Rockwell hardness	ASTM D785	R scale	101
Heat deflection temperature	ASTM D648	°C	108
Izod impact strength	ASTM D256	kg-cm/cm	2.1
Mold shrinkage	ASTM D955	%	1.2

**Table 2:** Pressure sensor specifications.

**Performance Characteristics**	
Ranges	0–30,000 psi
Accuracy	±0.5% FSO
Repeatability	±0.2% FSO
Mounting torque	500 inch–lbs. maximum
Maximum pressure	2 x full range or 35,000 psi (whichever is less)
Material in contact with pressure media	15- 5 PH stainless stell, DyMax™ coated
Weight	2 lbs
**Electrical Characteristics**	
Output	0 to 10 Vdc
Input Voltage	16 to 36 Vdc
Zero balance adjustment range	±15%

**Table 3:** Data acquisition module specifications.

**Analog Input**	
Channels	16
Resolution	16 bits
Max. sampling rate	200 kS/s
FIFO size	1024 samples
**Digital Input**	
Channels	8
Input voltage	Logic 0: 1.0 V max
	Logic 1: 2.0 V min

**Table 4:** Injection molding machine specifications.

CLF-60TX 600H-420D		
	Unit	Values
Screw diameter	mm	30
Theoretical injection volume	cm	141
Injection pressure	kg/cm^2^	2951
Injection rate	cm^3^/sec	115
Screw speed	rpm	325
Nozzle radius	mm	15
Clamping force	ton	60

**Table 5:** Experimental parameters with eight seconds of packing.

**Injection pressure (bar)**	**Clamping force (ton)**	**Packing pressure (bar)**	**Packing time (sec)**
170	30	15	8
**Run**	**Injection speed (mm/sec)**	**Switchover position (mm)**	**Melt temperature (°C)**
1	97.62	10	210
2	81.35		
3	65.08		
4	97.62	8	210
5	81.35		
6	65.08		
7	97.62	12	210
8	81.35		
9	65.08		
10	81.35	9	210
11	81.35	11	210

**Table 6:** Experimental parameters for three seconds of packing time with different amounts of clamping force.

**Injection pressure (bar)**	**Clamping force (ton)**	**Packing pressure (bar)**	**Packing time (sec)**
170	20, 30, 40	15	3
**Run**	**Injection speed (mm/sec)**	**Switchover position (mm)**	
1	81.35	8	
2		9	
3		10	
4		11	
5		12	

**Table 7:** Experiment parameters for three seconds of packing time.

**Injection pressure (bar)**	**Clamping force (ton)**	**Packing pressure (bar)**	**Packing time (sec)**
170	30	15	3
**Run**	**Injection speed (mm/sec)**	**Switchover position (mm)**	**Melt temperature (°C)**
1	97.62	8	210
2	81.35		
3	65.08		
4	97.62	9	210
5	81.35		
6	65.08		
7	97.62	10	210
8	81.35		
9	65.08		
10	97.62	10.5	210
11	81.35		
12	65.08		
13	97.62	11	210
14	81.35		
15	65.08		
16	97.62	11.5	210
17	81.35		
18	65.08		
19	97.62	12	210
20	81.35		
21	65.08		

**Table 8:** The experimental parameters without the adaptive adjustment system.

**Injection pressure (bar)**	**Clamping force (ton)**	**Packing pressure (bar)**	**Packing time (sec)**
170	40	15	3
**Cooling Time (sec)**	**Injection speed (mm/sec)**	**Switchover position (mm)**	**Melt temperature (°C)**
10	81.35	10	210

**Table 9:** The experiment parameters with the adaptive adjustment system.

**Injection pressure (bar)**	**Clamping force (ton)**	**Packing pressure (bar)**	**Packing time (sec)**
170	40	15	3
**Cooling Time (sec)**	**Injection speed (mm/sec)**	**Switchover position (mm)**	**Melt temperature (°C)**
10	81.35	8	210

## Data Availability

The data presented in this study are available on request from the corresponding author.
